# Mathematical Model Impact Analysis of a Real-Life Pre-exposure Prophylaxis and Treatment-As-Prevention Study Among Female Sex Workers in Cotonou, Benin

**DOI:** 10.1097/QAI.0000000000002535

**Published:** 2020-10-16

**Authors:** Lily Geidelberg, Kate M. Mitchell, Michel Alary, Aminata Mboup, Luc Béhanzin, Fernand Guédou, Nassirou Geraldo, Ella Goma-Matsétsé, Katia Giguère, Marlène Aza-Gnandji, Léon Kessou, Mamadou Diallo, René K. Kêkê, Moussa Bachabi, Kania Dramane, Christian Lafrance, Dissou Affolabi, Souleymane Diabaté, Marie-Pierre Gagnon, Djimon M. Zannou, Flore Gangbo, Romain Silhol, Fiona Cianci, Peter Vickerman, Marie-Claude Boily

**Affiliations:** a Medical Research Council Centre for Global Infectious Disease Analysis, Department of Infectious Disease Epidemiology, Imperial College London, London, United Kingdom;; b Département de Médecine Sociale et Préventive, Université Laval, Québec, Quebec, Canada;; c Axe Santé des Populations et Pratiques Optimales en Santé, Centre de Recherche du CHU de Québec–Université Laval, Québec, Quebec, Canada;; d Institut National de Santé Publique du Québec, Québec, Quebec, Canada;; e Dispensaire IST, Centre de Santé Communal de Cotonou 1, Cotonou, Bénin;; f École Nationale de Formation des Techniciens Supérieurs en Santé Publique et en Surveillance Épidémiologique, Université de Parakou, Parakou, Bénin;; g Service de Consultance et Expertise Nouvelle en Afrique (SCEN AFRIK), Cotonou, Bénin;; h Programme Santé de Lutte Contre le Sida (PSLS), Cotonou, Bénin;; i Faculté des Sciences de la Santé, Université d’Abomey-Calavi, Cotonou, Bénin;; j Centre National Hospitalier Universitaire HMK de Cotonou, Cotonou, Bénin;; k Université Alassane Ouattara, Bouake, Côte d'Ivoire;; l Faculté des Sciences Infirmières, Université Laval, Québec, Québec, Canada;; m Health Protection Surveillance Center, Dublin, Ireland; and; n Population Health Sciences, University of Bristol, Bristol, United Kindom.

**Keywords:** FSW, PrEP, TasP, Benin, modeling, prevention

## Abstract

Daily pre-exposure prophylaxis (PrEP) and treatment-as-prevention (TasP) reduce HIV acquisition and transmission risk, respectively. A demonstration study (2015–2017) assessed TasP and PrEP feasibility among female sex workers (FSW) in Cotonou, Benin.

**Setting:**

Cotonou, Benin.

**Methods:**

We developed a compartmental HIV transmission model featuring PrEP and antiretroviral therapy (ART) among the high-risk (FSW and clients) and low-risk populations, calibrated to historical epidemiological and demonstration study data, reflecting observed lower PrEP uptake, adherence and retention compared with TasP. We estimated the population-level impact of the 2-year study and several 20-year intervention scenarios, varying coverage and adherence independently and together. We report the percentage [median, 2.5th–97.5th percentile uncertainty interval (95% UI)] of HIV infections prevented comparing the intervention and counterfactual (2017 coverages: 0% PrEP and 49% ART) scenarios.

**Results:**

The 2-year study (2017 coverages: 9% PrEP and 83% ART) prevented an estimated 8% (95% UI 6–12) and 6% (3–10) infections among FSW over 2 and 20 years, respectively, compared with 7% (3–11) and 5% (2–9) overall. The PrEP and TasP arms prevented 0.4% (0.2–0.8) and 4.6% (2.2–8.7) infections overall over 20 years, respectively. Twenty-year PrEP and TasP scale-ups (2035 coverages: 47% PrEP and 88% ART) prevented 21% (17–26) and 17% (10–27) infections among FSW, respectively, and 5% (3–10) and 17% (10–27) overall. Compared with TasP scale-up alone, PrEP and TasP combined scale-up prevented 1.9× and 1.2× more infections among FSW and overall, respectively.

**Conclusions:**

The demonstration study impact was modest, and mostly from TasP. Increasing PrEP adherence and coverage improves impact substantially among FSW, but little overall. We recommend TasP in prevention packages.

## Abstract 2

Supplemental Digital Content is Available in the Text.

## INTRODUCTION

Pre-exposure prophylaxis (PrEP) and treatment-as-prevention (TasP) [rapid test-and-treat antiretroviral therapy (ART)] are 2 key HIV prevention interventions,^1–4^ with very high individual-level efficacy if there is adequate adherence.^5–8^ Clinical trials of PrEP among high-risk women in sub-Saharan Africa have shown mixed effectiveness results, partly due to poor adherence.^9–11^ After concerns of oral PrEP uptake and feasibility in real-life settings, several demonstration studies have been conducted among female sex workers (FSW),^12^ but to date, no modeling impact analyses assessing the impact of PrEP have reflected these real-life data.^13^ Despite this, PrEP is being offered and adopted across the region.^14^

As in many West African countries, the HIV epidemic in Benin has remained concentrated in key populations, with FSW disproportionately affected by HIV,^15,16^ exacerbated by the illegal status of sex work in Benin and other economic, structural, and social barriers to accessing health services. In 2015, the HIV prevalence in Benin was around 1% overall and 16% in FSW.^17^ Cotonou, the largest city in Benin, has a large population of professional FSW (pFSW) (ie, women for whom sex work constitutes most of their income) mainly brothel or street-based and part-time FSW (ie, sex work forms the minority of income) working in bars or restaurants.^17^ FSW often migrate from other areas of Benin or neighboring countries (eg, Nigeria, Togo, and Ghana)^18^ and are very mobile.^16^

Previous mathematical modeling suggests that in Cotonou commercial sex work may have contributed to more than 90% of all new HIV transmissions between 1980 and 1993 and 52% between 1993 and 2008, following the start of the effective SIDA 1/2/3 FSW prevention interventions in 1993.^19^ The HIV prevalence among professional FSW declined from 53% to 30% between 1993 and 2008, following increased consistency in condom use during commercial sex (from 30% to 88%).^16,20^ Despite this progress, the 2015 HIV prevalence in Cotonou among professional and part-time FSW remains high at 17% and 8%, respectively,^17^ compared with 2% in the general population in 2012.^21^

Currently, the national ART coverage levels are far below UNAIDS 2020 90-90-90 targets [90% of people living with HIV (PLHIV) diagnosed, 90% of diagnosed on ART, and 90% on ART virally suppressed], with 66% of women and 45% of men living with HIV estimated to be on ART in 2017.^22^ In 2015, only 24% of FSW living with HIV in Cotonou were on ART.^23^ The high HIV prevalence and low ART coverage in FSW may prevent Benin from achieving the UNAIDS goal of eliminating HIV/AIDS as a global health threat by 2030^21^; a continued focus and improvement in HIV prevention strategies for FSW remain important.

In late 2014, at a time when PrEP was unavailable and only individuals with T-cell CD4 count <500 cells/mL blood were eligible for ART,^24^ a 2-year demonstration project (henceforth called the “PrEP/TasP study”) was conducted to assess the “real-life” feasibility and usefulness of adding TasP and PrEP to the existing clinical, behavioral, and structural combination prevention package already offered to FSW in Benin.^25^ The study focused on pFSW from a designated catchment area in Cotonou, representing approximately 28% and 51% of all HIV-negative (HIV-) and HIV-positive (HIV+) pFSW in Cotonou and its surroundings.^16,17,25–27^ After tailored community outreach activities, preparedness, and adherence counseling by researchers and community workers with more than 20 years of HIV prevention experience among FSW, uptake, adherence, and retention to PrEP and TasP and self-reported sexual behavior were measured over 2 years (2015–2017) at quarterly visits in face-to-face interviews, with further pill counts and biological markers being used to measure adherence. Uptake (88% recruited of 290 eligible) and retention at study end (47% retained of 256 initiated) to PrEP were poorer than that for TasP (uptake: 96% of 110 and retention: 59% of 105), mostly because of migration and leaving sex work.^25^ Final visit viral suppression among TasP participants was 87% (N = 62), and tenofovir was detectable in blood plasma in 53% of 569 visits by PrEP participants; 2 seroconversions associated with poor adherence were observed in the PrEP arm.^25,28^

Our primary objective is to estimate the real-life impact of the PrEP/TasP study (including increased HIV testing and PrEP/TasP eligibility and initiation) compared with the standard of care that existed at the time. A secondary objective is to explore the impact of more optimistic PrEP and TasP scenarios. More specifically, we used a dynamical model of HIV transmission in Cotonou, calibrated with several rounds of FSW and clients integrated biobehavioral surveys (IBBS), household-based general population surveys, and the PrEP/TasP study results to estimate (1) the 2-year and 20-year impact of the 2-year PrEP/TasP study among pFSW, clients, and the wider population and (2) the impact of offering PrEP and/or TasP to all eligible pFSW in Cotonou and increasing intervention efforts (ie, improving coverage and/or adherence).

## METHODS

### PrEP/TasP Study Data

The PrEP/TasP study is registered with ClinicalTrials.gov (number NCT02237027), and the methodology and results have been previously published.^25^ PrEP/TasP study data were collected from the beginning of recruitment (October 4, 2014) until the end of follow-up (December 31, 2016). This model impact analysis involved no additional data collection.

### Model Structure

**FIGURE 1.**
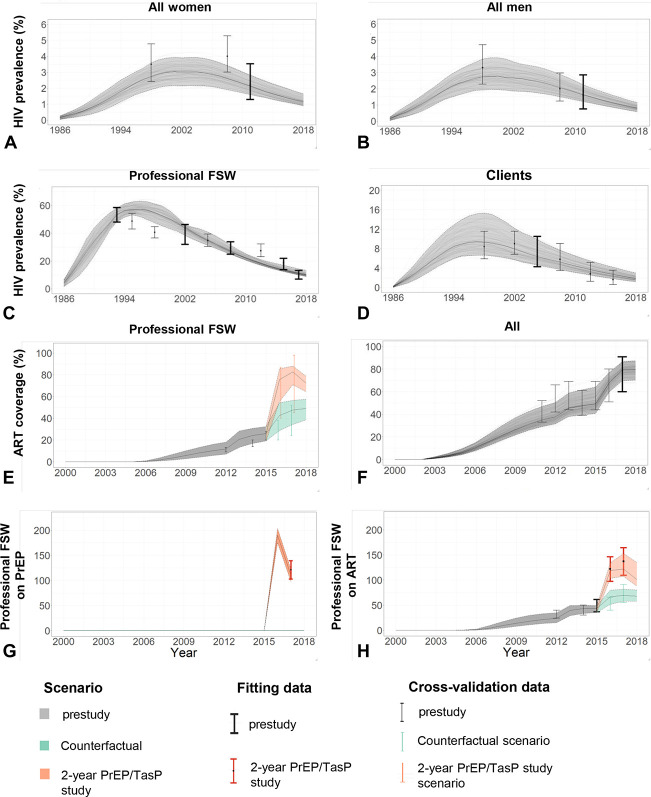
Model calibration: The results show model predictions from the 111 posterior parameter sets, in gray, green, and orange solid lines (median) and shaded regions (95% UI across all fits) representing prestudy, counterfactual (no PrEP or TasP), and 2-year PrEP/TasP study scenarios, respectively, compared with available fitting HIV prevalence and ART and PrEP coverage data shown in thick bars before 2015 (gray) and during the 2-year PrEP/TasP intervention study between 2015 and 2017 (orange). Results are shown for HIV prevalence (%) among (A) all women, (B) all men, (C) pFSW, (D) clients of FSW; ART coverage (%) among (E) HIV+ pFSW and (F) all HIV+; (G) number of pFSW on PrEP, and (H) on ART. Additional cross-validation data not used at the fitting stage are also presented in thin bars. Sections 6 and 7, Supplemental Digital Content 1, http://links.lww.com/QAI/B549 summarize additional fitting and cross-validation results, respectively.

We developed a dynamic compartmental model of sexual HIV transmission and interventions (PrEP, ART, and condom use) representing the open and growing heterosexual adult (15–59) population of Grand Cotonou (comprising Central Cotonou, Abomey-Calavi, and Seme-Kpodji), hereafter called “Cotonou.” The model population is stratified into 9 risk groups with differing sexual activity (ie, number and types of partners and condom use): professional and part-time FSW, clients of FSW, former FSW inside Cotonou and former FSW outside Cotonou, sexually active low-risk men and women, and not yet sexually active (NYSA) men and women (see Figure 1, Supplemental Digital Content 1, http://links.lww.com/QAI/B549). There is no age stratification.

Low-risk women may start sex work and join the professional or part-time FSW categories. Professional and part-time FSW are assumed to cease sex work and transition to the former FSW categories inside or outside of Cotonou (reflecting the proportion who are foreign born) at rates based on their average duration in sex work. Similarly, sexually active low-risk men become clients of FSW and return to the low-risk male population after a fixed duration of being a client. NYSA women and men transition to the low-risk sexually active population at rates determined by their average age at sexual debut. Transitions between risk groups are independent of stage of infection and HIV care state. Between clients and professional and part-time FSW, there are commercial (generally higher partner change rate and condom use) and noncommercial (generally more sex acts per partner and less condom use) partnerships. All other sexually active groups in Cotonou form only noncommercial partnerships (see Figure 1, Supplemental Digital Content 1, http://links.lww.com/QAI/B549). Although represented in the model, NYSA women and men, and former FSW outside Cotonou, do not form sexual partnerships. The number of partnerships between different risk and gender groups is balanced at each time step (details in Section 2, see Materials, Supplemental Digital Content 1, http://links.lww.com/QAI/B549).^29^

New individuals join all groups except clients and former FSW. To represent the FSW migrant population, some pFSW join as HIV+ reflecting HIV prevalence in their countries of origin; all other groups join as HIV-. Individuals leave the modeled population because of aging and HIV-related and HIV-unrelated mortality.

**FIGURE 2.**
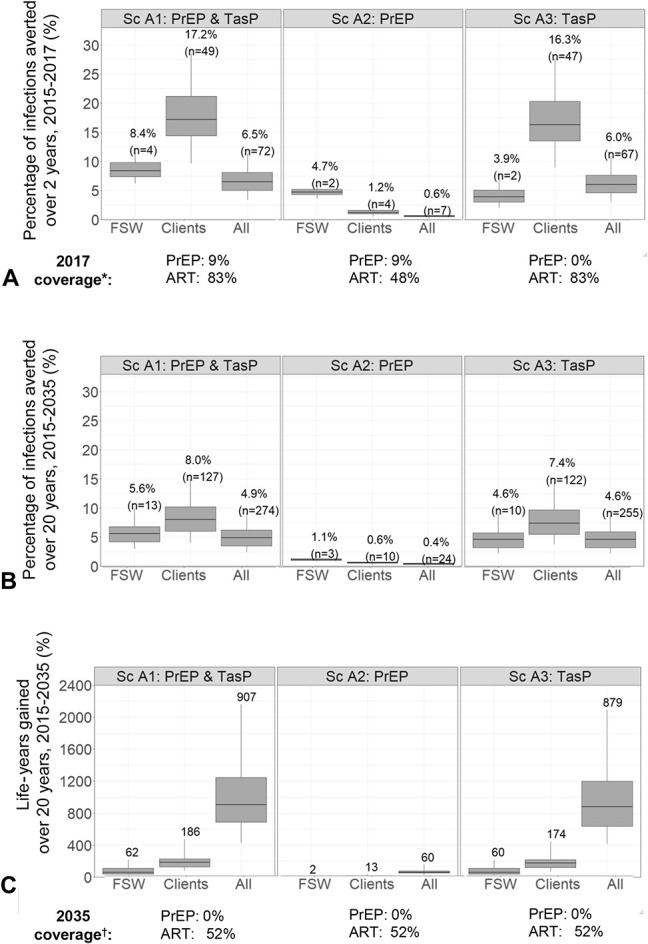
Impact of the 2-year PrEP/TasP study for PrEP and TasP combined (scenario A1), PrEP alone (Sc A2), or TasP alone (Sc A3), among pFSW (“FSW”), clients, and the whole population (“All”) in terms of percentage of HIV infections prevented over (A) 2 years and (B) 20 years, and C) life-years gained (LYG) over 20 years, compared with counterfactual scenario (no PrEP; ART coverage 49% and 52% in 2017 and 2035, respectively). Box plots represent the median impact (central horizontal line), 25th to 75th percentiles (box), and 95% UI (whiskers) across 111 parameter sets. Labels in panels A and B show the median percentage (and number in brackets) of infections prevented; panel C shows median LYG. The PrEP and ART coverage represent median posterior values across all model fits for each scenario as shown in Table 3. *The median coverage of HIV- and HIV+ for PrEP and ART, respectively, at the end of the 2-year study. †Median coverage in 2035, 20 years after the start of PrEP/TasP study.

The model represents HIV disease progression stratified by CD4 cell count levels and different levels of engagement in the care and treatment cascade (see Figure 2, Supplemental Digital Content 1, http://links.lww.com/QAI/B549). Untreated, undiagnosed HIV-positive individuals progress through a highly infectious primary infection stage, followed by 4 longer disease stages (CD4 >500, CD4 350–500, CD4 200–350, and CD4 <200 cells/µL); the latter 3 stages incur HIV-related mortality, and the primary and CD4<200 stages incur higher infectiousness.

HIV- pFSW can initiate PrEP and move into perfectly adherent, intermediate- adherent, or nonadherent categories at fixed proportions, each with different efficacy (per sex act HIV transmission reduction), and from which they can move into lower adherence categories or drop out. Only pFSW can initiate PrEP. Undiagnosed HIV+ individuals are tested for HIV at a rate dependent on the risk group and stage of infection and move to the diagnosed off ART category, who experience the same disease progression. Diagnosed individuals initiate treatment at a rate dependent on the risk group, stage of infection, and calendar year (reflecting changes in eligibility criteria and ART provision). Compared with untreated individuals, PLHIV on ART have slower rates of disease progression and reduced HIV-related mortality. ART effectiveness depends on the proportion of PLHIV on ART that are virally suppressed, which varies by the risk group. Individuals can cease using ART, whereby disease progression occurs at the same rate as for undiagnosed individuals unless they re-initiate treatment.

Sexually active HIV- individuals become infected with HIV at a per capita force of infection dependent on the frequency of commercial and noncommercial partnerships, number of sex acts per partnership with and without condoms, PrEP status and adherence, HIV prevalence among partners, per-act HIV infectivity that depends on partners' disease stage, ART status and viral suppression, and efficacies of different interventions. Condom use and ART coverage increase over time and vary by the risk group reflecting data from Cotonou.^16,17,20,21,26,30–34^

The model is expressed as a system of ordinary differential equations solved numerically from 1986 to 2035 using the LSODA algorithm with a variable time step,^35^ The model was coded in R, using “odin”,^36^ a wrapper around the deSolve package.^37^ Additional details are provided in the Supplemental Materials.

### Model Calibration

The model was calibrated within a Bayesian framework, as previously described.^38^ Using various data sources we defined prespecified plausible prior ranges for 87 demographic, behavioral, biological, and intervention model parameters (Table 1, see Table 1, Supplemental Digital Content 1, http://links.lww.com/QAI/B549) and 31 different demographic, epidemiological, and intervention fitting targets before and after the PrEP/TasP study (Table 2).

**TABLE 1.** Summary of Key Model Parameter Values and Prior Ranges

Parameters	Date: Value/Prior range*	Source and Comments
A) Demographic		
Total population size of Grand Cotonou (N)	1979: 286,114	^42^
Population growth rate (year^−1^)	1979–1992: 0.059	^39–42^
	1993–2002: *0.048–0.058*	
	2002–2035: 0.027	
Percentage of women who are pFSW at seeding (%)	1986: *0.24*–*0.72%*	^27,39–42,46^
Percentage of men who are clients at seeding (%)	1986: *7–30%*	^18,39–42,44^
Rate of stopping sex work (year^−1^)	*0–0.55*	^16,17,20,26^
B) Sexual behaviour		
Commercial partnerships per pFSW (year^−1^)	1986–1993: *192–1277*†	^16,17,20,26^
	2005–2036: *81*–*562*	
Commercial partnerships per client (year^−1^)	1986–1998: *8–32*	^18,31–33^
	2002–2035: *11–20*	
Sex acts per commercial partnership, pFSW, and client (year^−1^)	*1–3*	^16–18,20,26,31–33^
Condom use pFSW and client in commercial sex acts (%)	1986–1993: *18–33%*	^16–18,20,26,31–33^
	2008–2035: *86–99%*	
Noncommercial partnerships per pFSW (year^−1^)	*0.31–0.86*	^16,17,20,26^
Noncommercial partnerships per client (year^−1^)	*1.6–3.3*	
Sex acts per noncommercial partnership, pFSW, and client (unit)	1986–2002: *13–20*	^16,17,20,26,31–33^
	2015–2035: *38–60*	
Condom use pFSW and client in noncommercial sex acts (%)	1986: *0%*	^16,17,20,26,31–33^
	2002–2035: *19–62%*	
C) Biological		
HIV transmission probability male to female per sex act (%)	*0.06–0.109%*	^51^
Relative risk of transmission female to male	*0.5–2*	^51^
Relative risk during primary stage	*4.5–18.8*	^51^
Relative risk during CD4 < 200 stage	*4.5–11.9*	^51^
Condom efficacy: Reduction in HIV transmission probability per sex act (%)	*58*–*95%*	^19^
D) Intervention		
Probability tested in the last yr (pFSW) (%)	1986–2005: 0%	Annual testing rate calculated as function of probability of testing in the last yr^16,17,20,21,26,30–34,45,47^
	2013–2015: 65%	
	2015–2035: 68%	
Probability tested in the last yr (low-risk women)	1986–2001: 0%	
	2006: 14%	
	2008: 21%	
	2011–2035: 33%	
Probability tested in the last yr (low-risk men)	1986–2001: 0%	
	2006: 10%	
	2011–2035: 6%	
ART eligibility	2005: CD4 < 200 (all groups)	In scenarios involving TasP (Table 2), all HIV+ pFSW eligible for ART from 2015 onward.^21^
	2012: CD4 < 350 (all groups)	
	2015: CD4 < 500 (all groups)	
	2016: CD4 < 500 (professional FSW)	
	2016: Any CD4 (all other groups)	
ART initiation rate (among diagnosed pFSW) (year^−1^)	*0.25*–*6*	^23^
ART initiation rate (among all other groups diagnosed) (year^−1^)	*6–12*	^23^
Proportion of those on ART virally suppressed (all groups) (%)	1986–2015 (pFSW) and 1986–2035 (all other groups): *42–85%*	^25,66^
ART dropout rate (all groups) (year^−1^)	*0.023–0.11*	This rate also applies to pFSW on ART recruited to TasP^21^
PrEP/TasP study testing rate	2015–2017: *0.5–2.0*	This rate is added to the annual testing rate for pFSW^25^
(E) 2-year PrEP study arm (2015–2017)		
PrEP initiation rate (year^−1^)	2015–2016: *0.2–0.4*	^25^
Distribution by adherence levels (%)	Low: 46.6%	Based on the observed proportion of blood plasma samples by tenofovir concentration (ng/mL) among FSW on PrEP in the study^25,28^
	Intermediate: 16.5%	
	High: 36.9%	
PrEP efficacy per adherence level: Reduction in HIV acquisition probability per sex act (%)	Low: 0%	Derived from Donnell et al 2014 and expert opinion. Average PrEP effectiveness^25^ = 41%‡
	Medium: *30–49%*	
	High: *90–95%*	
PrEP dropout rate (year^−1^)	*0.2–0.5*	Does not include FSW leaving sex work or leaving Cotonou^25^
(F) 2-year TasP study arm (2015–2017)		
TasP initiation rate (year^−1^)	*0.5–5*	^25^
Proportion pFSW on ART virally suppressed (%)	*75–87%*	^25,66^
ART efficacy: Reduction in HIV transmission probability per sex act if virally suppressed (%)	*96–99%*	^5^

(A) demographic, B) sexual behavior, C) biological, D) intervention, E) PrEP study arm, and (E) TasP study arm parameters. Full parameter list in Table 1, Supplemental Digital Content 1, http://links.lww.com/QAI/B549.

* Estimates are fixed; ranges (in italics) were sampled in the Latin Hypercube. If no date is given, it was fixed at the value throughout the epidemic.

† Between sampled yr, parameter values are linearly interpolated.

‡ Calculated as the sum of product (average adherence × efficacy) across all adherence levels.

**TABLE 2.** Fitting Outcomes

Fitting Outcomes	Year (Start)	Target Range/Estimate	Source
A) Prestudy fitting			
Stage 1 (1986–2015): Demographic			
Total population size of sexually active adult population of Grand Cotonou (N)	1992	343,705–465,013	^39–42^
	2002	579,325–783,793	
	2013	776,076–1,049,985	
	2020	959,418–1,298,036	
	2030	1,210,305–1,637,471	
Percentage of women who are pFSW (%)	All	0.19–0.72	^27,39–42,46^
Number of pFSW (N)	2012	889–1391	^27^
Percentage of women who are active FSW (professional + part-time) (%)	All	0.48–1.4	^27,39–42,46^
Percentage of men who are clients (%)	All	7.4–30	^18,39-42,44^
Percentage of women who are NYSA (%)	All	7.9–20	^30,45,47^
Percentage of men who are NYSA (%)	All	7–17	^30,45,47^
Stage 2 (1986–2015): Epidemiological			
HIV Prevalence pFSW (%)	1993	48–58	^16,17,20,26,64^
	2002	32–46	
	2008	25–34	
	2015	14–22*	
	2017	7–13*	
HIV Prevalence clients (%)	2002	6.8–12.0	^31–33^
HIV Prevalence all women (%)	2011	1.3–3.5	^47^
HIV Prevalence all men (%)	2011	0.75–2.9	^47^
HIV Incidence rate pFSW (% infected per person-yr)	2015	0–3	^48^
Stage 2 (1986–2015): ART coverage			
Professional HIV-infected FSW on ART (N)	2015	42–56	^23^
Men and women on ART combined (N)	2017	8524–18,273	^66^
ART coverage of men and women combined (%)	2011	33–52	^21,66^
ART coverage of men and women combined (%)	2017	60–91	^21,66^
Ratio of women to men on ART	2017	1.5–3.0	^66^
B) PrEP/TasP study scenario (2015–2017)			
Person-yr on PrEP (yr)	2015–2017	250	^25^
PrEP initiations (N)	2015	256	
Number on PrEP at end of study (N)	2017	121	
TasP initiations (N)	2015	105	
All pFSW on ART at end of first yr (N)	2016	122	
All pFSW on ART at end of study (N)	2017	137	

Outcomes and target range or estimate used for the A) pre-PrEP/TasP study model fitting (before 2015) and B) the PrEP/TasP study scenario model fitting (2015–2017).

* Parameter sets that produced model outcomes meeting at least one of the empirical estimates of HIV prevalence among pFSW in 2015 and 2017 were accepted.

Benin census data,^39–42^ the UN World Population Prospect,^43^ and studies estimating the size of pFSW and client populations^18,27,44–46^ informed demographic parameters and targets. Sexual behavior parameters were informed by several rounds of pFSW and client IBBS in Cotonou and 3 household-based general population surveys.^16,17,20,26,30–33,45,47^ Cotonou pFSW HIV incidence and biological parameters for HIV natural history and infectivity were sourced from published literature.^48–63^ ART has been available in Benin since 2002.^21^ Data on HIV prevalence by risk group, HIV testing, ART uptake and coverage, and adherence (viral suppression) and dropout among pFSW and other groups were also derived from the IBBS,^16,17,20,26,31–33,64^ household-based surveys in Cotonou,^30,45,47^ and government reports.^21,23,34,65,66^ PrEP was unavailable in Cotonou before the PrEP/TasP study. The study provided biologically measured estimates of drug adherence (PrEP: up to 5 repeat tenofovir blood plasma concentration measures per individual; TasP: viral load),^28^ dropout rates (derived from visit attendance over the 2 years), and TasP and PrEP initiation and coverage.^25^ PrEP efficacies by the adherence level (Table 1E) were derived from Donnell et al.^9^

### Prestudy Model Fitting

The model was first fitted prestudy (Table 2A) in 2 stages using Latin Hypercube Sampling.^67^ In stage 1, we sampled our prespecified demographic priors to form 7000 parameter sets and retained those that produced model estimates meeting the 11 prespecified demographic targets (442 parameter sets retained) (Table 2). In stage 2 (epidemiological), the posterior ranges of these 442 parameter sets were sampled 79 million times, retaining those that produced model outcomes meeting all 25 fitting targets before 2015 (111 parameter sets retained). These modeled epidemic trajectories, therefore, reflect the empirical data in Cotonou. Some fitting targets (eg, men and women on ART combined) are after 2015, but these were insignificantly impacted by the PrEP/TasP study; hence, we called this stage “prestudy” for clarity. Additional available data not used for model fitting were used for cross-validation (see Section 5, Supplemental Digital Content 1, http://links.lww.com/QAI/B549).

### Scenarios

We used the final posterior parameter sets from the pre-PrEP/TasP intervention fitting to simulate the 2-year PrEP/TasP study scenario, various long-term extension/scale-up intervention scenarios, and the counterfactual scenario, summarized in Table 3.

**TABLE 3.** Short-Term and Long-Term Scenarios Investigated

Scenario		Increased HIV Testing Among FSW*	PrEP Among FSW		ART Among FSW		Median Coverage Across 111 Fits (%)		
			Initiation rate†	Adherence	Eligibility Criteria (from 2015)	TasP Initiation & Dropout rates†		2017	2035
C0	Counterfactual (no PrEP or TasP)	—	—	—	CD4 < 500	—	PrEP	0%	0%
							ART	49%	52%
PrEP/TasP study scenarios (2015–2017)‡									
A1	Observed PrEP/TasP study	As observed	As observed	As observed (Table 1E)	Any CD4 (until 2017)§	As observed	PrEP	9%	0%
							ART	83%	53%
A2	PrEP study arm alone	As observed	As observed	As observed	CD4 < 500	—	PrEP	9%	0%
							ART	As counterfactual	
A3	TasP study arm alone	As observed	—	—	Any CD4 (until 2017)§	As observed	PrEP	As counterfactual	
							ART	83%	53%
Long-term 20-year interventions scenarios (2015–2035)									
Long-term PrEP alone¶									
B1	PrEP extension║	As observed	As observed	i) As observed ii) perfect	CD4 < 500	—	PrEP	24%†	20%
							ART	As counterfactual	
B2	PrEP scale-up║	As observed	2× higher than observed; allow re-initiations	i) As observed ii) perfect	CD4 < 500	—	PrEP	37%	47%
							ART	As counterfactual	
Long-term TasP alone									
B3	TasP extension	As observed	—	—	Any CD4	As observed	PrEP	As counterfactual	
							ART	83%	81%
B4	TasP scale-up	As observed	—	—	Any CD4	Initiation 2× higher and dropout 4× lower than observed	PrEP	As counterfactual	
							ART	89%	88%
Long-term PrEP and TasP combined¶									
B5	PrEP extension║ + TasP scale-up	As observed	As observed	i) As observed ii) perfect	Any CD4	Initiation 2× higher and dropout 4× lower than observed	PrEP	As B1	
							ART	As B4	
B6	PrEP scale-up║ + TasP scale-up	As observed	2x higher than observed; allow re-initiations	i) As observed ii) perfect	Any CD4	Initiation 2× higher and dropout 4× lower than observed	PrEP	As B2	
							ART	As B5	

A) the 2-year PrEP/TasP study and (B) various extended and scaled up 20-year PrEP and TasP interventions.

PrEP dropout rate kept constant at rate observed in study. Prior ranges for all parameters are given in Section 4, Supplemental Digital Content 1, http://links.lww.com/QAI/B549.

* Increased testing among pFSW applied over the duration of the intervention (PrEP/TasP study scenarios: 2015–2017; long-term scenarios: 2015–2035).

† PrEP and TasP initiation rates among HIV− and HIV+ pFSW (PrEP/TasP study scenarios as observed: 2015–2016; long-term scenarios: 2015–2035).

‡ PrEP and TasP were available between 2015 and 2017, and impact measured both over 2 years (2015–2017) and over 20 years (2015–2035).

§ Eligibility returns to CD4<500 in 2017.

║ All 20-year scenarios involving PrEP are simulated twice: either assuming adherence as observed or assuming all FSW adhere perfectly.

¶Note long-term PrEP initiation rate assumed constant from 2015 onward (c.f. in the study where initiation only lasted 1 yr: 2015–2016).

### PrEP/TasP Study Scenario Model Fitting (2015–2017)

We obtained this scenario by fitting the model to the 2-year PrEP/TasP targets, reflecting the observed study data (adherence, retention, and coverage). First, we defined prior ranges for 6 PrEP and TasP parameters (Table 1E, F). For each of the 111 parameter sets retained after the prestudy fitting, we sampled the ranges of the 6 PrEP/TasP parameters 100 times separately and retained the set that produced model results best matching (using a least squares method) the study targets (Table 2B). PrEP and TasP recruitment lasted only for 1 year (2015–2016), as observed.

This specific scenario assumed that HIV testing, PrEP, and ART eligibility returned to the prestudy levels after 2 years (at study end), that is, PrEP was unavailable, and ART eligibility for pFSW returned to CD4<500 cells/µL (pFSW recruited to TasP remained on ART poststudy). We simulated the HIV epidemic from 1986 to 2035 using these assumptions (PrEP/TasP study scenario A1, Table 3). The PrEP and TasP arms of the study were also simulated alone (scenarios A2 and A3, respectively) to estimate the relative impact of each.

### Long-Term 20-Year Intervention Scenarios (2015–2035)

We also simulated more optimistic long-term PrEP and/or TasP “extension” and “scale-up” scenarios, which offered interventions to all eligible pFSW for 20 years (2015–2035) instead of 2 years. The “extension” scenarios maintained over 20 years the same HIV testing, PrEP/ART initiation, and dropout rates as the PrEP/TasP study scenario. “Scale-up” scenarios assumed faster initiation and/or slower dropout rates (Table 3) within limits of what was deemed realistic. We assumed no condom migration, as observed.^68^

In all long-term intervention scenarios, pFSW HIV testing equaled the PrEP/TasP study rate. When modeling PrEP alone, we assume that ART eligibility remained at CD4<500 cells/µL and that PrEP daily adherence patterns were either (1) equal to the 2-year study or (2) “perfect” adherence. For long-term TasP intervention scenarios, all HIV+ pFSW are eligible for ART for 20 years.

### Counterfactual Scenario

Our counterfactual scenario simulates the HIV epidemic over 1986–2035 without PrEP or TasP among pFSW. We assumed that all parameters (including HIV testing, ART initiation, and dropout rates) remain constant from 2015 onward, PrEP never becomes available, and ART eligibility remains at CD4<500 cells/µL (reflecting guidelines in 2015) for pFSW until 2035. Despite all HIV+ key populations later becoming eligible for ART,^25^ CD4<500 was the “standard of care” at the time of the study and was therefore used as the counterfactual to estimate the impact of TasP (the combined effect of increased ART eligibility, initiation, and HIV testing), to answer our primary modeling objective. We ensured the number of pFSW on ART predicted in the model counterfactual in 2017 was consistent with the observed number within the Beninese health system.

Finally, given that ART eligibility was extended to all HIV+ FSW after the study, we repeated the analyses with a second counterfactual assuming reflecting this. This facilitates understanding the impact of long-term TasP from increased ART initiation and HIV testing but not the change in eligibility. These results are shown in the Materials Section S9, Supplemental Digital Content 1, http://links.lww.com/QAI/B549.

### Impact Outcomes

For each model fit and intervention scenario, we estimated the annual number of incident HIV infections and life-years lived (the number of individuals at each mid-year) for each risk group. We calculated HIV infections prevented and life-years gained over 2-year (2015–2017) and 20-year (2015–2035) periods by comparing each outcome between the intervention and counterfactual scenarios, among pFSW, clients, and the whole population. We derived the proportion of HIV infections prevented as a percentage of the number of infections in the counterfactual scenario within the relevant time horizon. We report the median impact and the 2.5th and 97.5th percentile [95% uncertainty interval (UI)] from all model fits.

### Ethical Approval

The PrEP/TasP demonstration study protocol received approval from the Ethics Committee of the CHU de Québec–Université Laval, Québec, Canada and the Benin National Ethics Committee for Health Research. All participants provided written consent in a free and informed manner and could decline or withdraw from the study at any time. Participants received a financial compensation of 4000 FCFA (about $8) at each visit to compensate for their travel cost and the time they devoted to the study. For this model impact analysis, we received approval from the Imperial College Research Ethics Committee (ICREC reference: 18IC4641) to perform our secondary data analysis on data from the PrEP/TasP demonstration study (2014–2017). This model impact analysis involved no additional data collection.

## RESULTS

### Model Calibration

Model predictions based on the 111 posterior parameter sets (Fig. 1) agreed well with available HIV prevalence and ART fitting data, other demographic, epidemiological, and intervention outcomes (Table 2) and HIV prevalence and deaths cross-validation data (see Section 5, Supplemental Digital Content 1, http://links.lww.com/QAI/B549). The model posterior HIV prevalence in 2017 among pFSW was estimated at 11% (95% UI 10–14), with long-term decreasing prevalence in all groups. From 2015 to 2017, the model also replicated closely the observed numbers of pFSW on PrEP and ART (including TasP) in the 2-year study scenario, and the observed number on ART but not on TasP in the counterfactual scenario (Fig. 1H). The median posterior coverage of PrEP and TasP at study end was 9% and 34%, respectively; total ART coverage including those not on TasP was 83%.

### Impact of the 2-Year PrEP/TasP Study Evaluated Over 2 and 20 Years

Figure 2 shows the estimated impact of the 2-year PrEP/TasP study (Sc.A1) over 2 (Fig. 2A) and 20 years (Figs. 2B, C). Model results suggest that between 2015 and 2017 the 2-year PrEP/TasP study prevented a median of 8% (95% UI 6–12), 17% (95% UI 10–29), and 7% (95% UI 3–11) of new HIV infections among all pFSW, clients, and in the whole population, respectively, representing 4 (2–5), 49 (27–91), and 72 (41–119) infections prevented (Fig. 2A).

Over 20 years, despite the lower percentages of HIV infections prevented (as intervention efforts ceased after study completion), the 2-year study prevented 13 (6–26), 127 (60–262), and 274 (142–608) new infections over 20 years and saved 62 (25–219), 186 (80–473), and 907 (430–2157) life-years among pFSW, clients, and overall between 2015 and 2035, respectively (Figs. 2B, C).

When measured over both 2 and 20 years (Figs. 2A, B), the PrEP study arm prevented a greater percentage of infections among pFSW than clients or overall, whereas the TasP arm prevented more infections among clients than pFSW. Compared with PrEP alone, the TasP arm prevented 4×, 13×, and 11× more infections and gained 32×, 13×, and 15× more life-years among pFSW, clients, and overall, respectively, over 20 years (Figs. 2A–C).

### Impact of 20-Year Long-Term PrEP or TasP Interventions Alone Evaluated Over 20 Years

**FIGURE 3.**
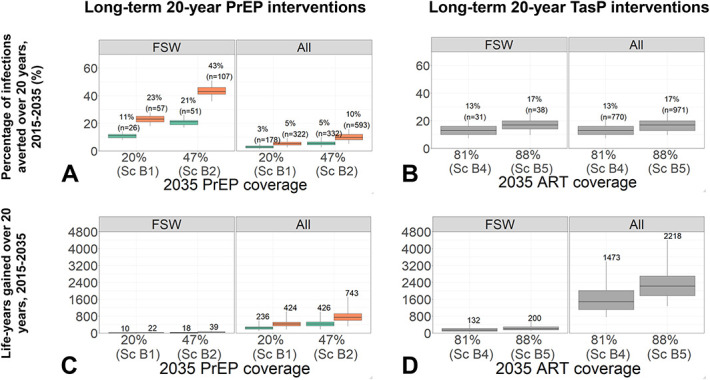
Impact of long-term 20-year PrEP (A, C) or TasP (B, D) intervention scenarios separately in terms of the percentage of infections prevented (A, B) and life-years gained (C, D) among pFSW (“FSW”) and the whole population (“All”), compared with the counterfactual scenario (no PrEP; 52% HIV+ pFSW ART coverage in 2035). Box plots represent the median impact (central horizontal line), 25th to 75th percentiles (box), and 95% UI (whiskers) across 111 parameter sets. The PrEP and ART coverages represent median posterior values in 2035 (among HIV- and HIV+ pFSW, respectively) across all model fits for each scenario as shown in Table 3. Adherence to PrEP assumed either as observed in the 2-year study (green), or perfect (orange). Labels in panels A and B show median percentage (and number in brackets) of infections prevented; panels C and D show median life-years gained.

Figure 3 compares the long-term impact of extending and scaling-up PrEP (Figs. 3A, C) and TasP (Figs. 3B, D) interventions separately (scenarios in Table 2), by increasing adherence (PrEP alone) and initiation (PrEP and TasP) and decreasing dropout (TasP alone). Assuming the observed study PrEP dropout rate (including migration), extending PrEP for 20 years achieved a modest 20% coverage in 2035 among HIV- pFSW (Sc.B1), preventing 11% (8–14) and 3% (1–5) of new infections over 20 years among pFSW and in the whole population, respectively, if adherence remains as observed in the study. Compared with PrEP extension (Sc.B1), the percentage of infections prevented (and life-years gained) over 20 years among pFSW and overall doubled by either increasing 2035 PrEP coverage to 47% (Sc.B2) or assuming perfect PrEP adherence and increased by 3–4 fold to 43% (36–51) and 10% (5–16) in pFSW and overall, by achieving both.

By contrast, extending TasP (Sc.B3) achieved a 2035 ART coverage of 81% (compared with 52% in the counterfactual) and prevented 13% (7–22) infections among pFSW and overall (Fig. 3B). Scaling-up TasP to 88% coverage (Sc.B4) prevented 17% (10–27) infections among pFSW and overall. TasP extension/scale-up has a 3–4× higher impact than the 20-year impact of the 2-year TasP study arm (Sc.A3). Although PrEP and TasP extensions each prevented similar infections among pFSW (11–13%), the population-level impact was ∼4× higher for TasP (13% vs. 3%).

At 81% ART coverage, 132 (58–419) and 1473 (758–3391) life-years were gained in pFSW and overall, respectively, and 200 (95–523) and 2218 (1274–4407) at 88% coverage. Compared with the most optimistic PrEP scenario (47% coverage; perfect adherence), TasP extension/scale-up gained 3–5× and 2–3× more life-years among pFSW and overall, respectively.

### Impact of 20-Year Combined PrEP and TasP Scale-Ups Evaluated Over 20 Years

**FIGURE 4.**
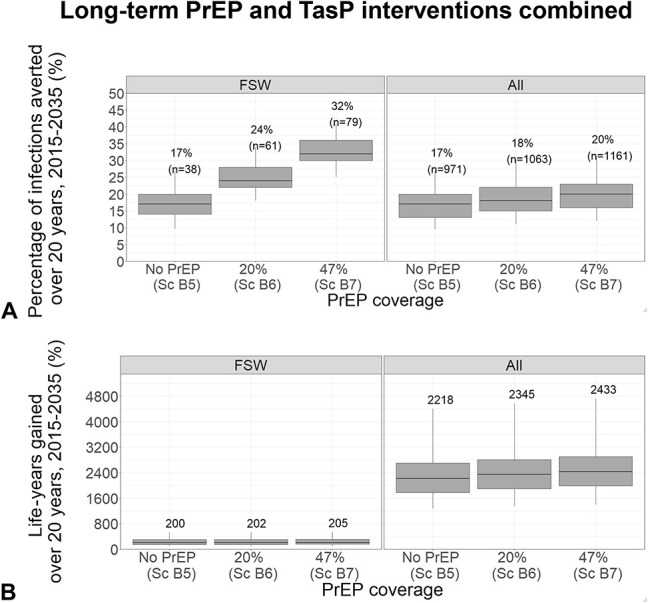
Impact of combined 20-year PrEP interventions and TasP scale-ups (88% coverage HIV+ pFSW in 2035), in terms of the percentage of infections prevented (A) and life-years gained (B) among pFSW (“FSW”) and the whole population (“All”), compared with the counterfactual scenario (no PrEP; 52% HIV+ pFSW ART coverage in 2035). Box plots represent the median impact (central horizontal line), 25th to 75th percentiles (box), and 95% UI (whiskers) across 111 parameter sets. PrEP coverages represent median posterior values in 2035 (among HIV- pFSW) across all model fits for each scenario as shown in Table 3; adherence assumed as observed in 2-year study. Labels in panel A show median percentage (and number in brackets) of infections prevented; panel B shows median life-years gained.

A combined PrEP and TasP scale-up to 47% of HIV- and 88% of HIV+ pFSW, respectively, by 2035 (Sc.B6) prevented 32% (25–41) of infections among pFSW and 20% (12–31) overall. Compared with TasP scale-up alone, PrEP and TasP scale-up combined corresponded to around twice the infections prevented among pFSW and 18% higher overall (Fig. 4A); however, this only increased life-years gained by 2.5% among pFSW and 10% overall (Fig. 4B).

## DISCUSSION

Our study is the first model impact analysis of PrEP and TasP among FSW reflecting observed real-life uptake, adherence, and retention data.^25^ We estimated that the recent PrEP/TasP demonstration study (2015–2017) in Cotonou prevented 8% and 6% of HIV infections among pFSW over 2 and 20 years, respectively, and 7% and 5% in the whole population. Over 20 years, the study gained an estimated 62 and 907 life-years among pFSW and overall, respectively. Unsurprisingly, the PrEP arm had the greatest impact among pFSW, whereas the TasP arm had the greatest impact among clients due to directly reducing transmission from pFSW to clients, with the TasP arm preventing 16% and 7% of infections among clients over 2 and 20 years, respectively. Although the study impact was modest, further work will elucidate whether it was cost-effective.

Overall, there was a greater impact (in terms of infections prevented and life-years gained) from the TasP arm compared with PrEP for several reasons. First, we assumed that PrEP was unavailable poststudy because it was not recommended by the national guidelines, whereas we assumed those on TasP continued treatment (as observed).^25^ Second, TasP had a higher coverage (34% of HIV+) than PrEP (9% of HIV-) after 2 years among pFSW in Cotonou due to higher uptake and retention; FSW on PrEP had a high dropout due to migration and leaving sex work. Third, there was better adherence and thus higher preventative efficacy for TasP than PrEP (averaging 81% pFSW on ART virally suppressed vs. 37% of visits with perfect PrEP adherence).^25,28^ Finally, TasP reduces mortality,^69^ and thus gaining life-years among HIV+ pFSW.

Similar to the 2-year study results, we predicted a greater impact for the extended and scaled-up TasP interventions than PrEP. Compared with 20-year TasP extension, PrEP extension prevented similar infections among pFSW (11–13%) but 4-times fewer infections overall; even PrEP scale-up to 47% HIV- pFSW with perfect adherence had less impact overall. Long-term TasP always gained more life-years than PrEP. Increasing PrEP coverage beyond 50% would be unfeasible according to country-level experts, mainly due to health care and funding constraints, and high observed turnover of pFSW in Cotonou.^17,26^ With TasP scale-up to 88% HIV+ pFSW in 2035, our analysis suggests little additional impact was gained by simultaneously increasing PrEP coverage to 47%.

In a sensitivity analysis (see Materials S9, Supplemental Digital Content 1, http://links.lww.com/QAI/B549) we explore a second counterfactual, whereby ART is available for all HIV+ pFSW from 2015 (as in long-term TasP scenarios). We found that removing the added effect of increased eligibility reduces the impact of TasP (in infections prevented) by around 30%; however, TasP extension still prevents more infections overall than PrEP, unless 47% coverage and perfect adherence can be achieved. All long-term TasP scenarios still gain more life-years among all groups than any long-term PrEP scenario.

Finally, our results suggest that efforts to improve PrEP adherence may be as beneficial as increasing coverage. For example, achieving perfect adherence with a PrEP coverage of 20% had a similar impact as increasing PrEP coverage to 47% with study level adherence. The observed PrEP adherence in the demonstrated study was despite the important components of outreach and adherence counseling. However, long-acting PrEP (injectable cabotegravir) was recently shown to have a high efficacy among men who have sex with men and transgender women,^70^ reducing the barriers to adherence with daily pills. Although long-acting PrEP results for women are not yet available,^71^ if similar adherence can be achieved, our perfect PrEP adherence scenario may be interpreted as the impact of long-acting PrEP.

Low-PrEP coverage in the study was due to poor retention and high mobility—53% of PrEP study participants dropped out and of these 58% left either Cotonou or sex work.^25^ The effectiveness of PrEP interventions among pFSW may be improved by identifying factors associated with better adherence and retention.

### Comparison With Other Studies

Although this is the first impact analysis of PrEP among FSW reflecting real-life demonstration study data on uptake, adherence, and retention, our results are comparable with previous studies that explore similar parameter values, despite differences in epidemiological conditions across settings. Assuming 30% annual PrEP uptake and 40% effectiveness, Bekker estimated ∼8% infections prevented over 10 years among FSW in South Africa.^72^ We estimate that with similar annual PrEP uptake (26%) and average effectiveness (41%, Table 1), our PrEP extension scenario (Sc.B1) prevented 11% (8–14) of infections in pFSW over 20 years. The slight differences are partially explained by the 10% condom use reduction in South Africa, whereas no condom migration was observed in pFSW in Cotonou.^68^ Low et al^73^ estimated that providing TasP to FSW in Burkina Faso to an ART coverage of 79% (compared with 37% in their base-case scenario) would prevent 15% of infections over 20 years overall. This result is comparable with our TasP extension scenario (Sc.B3) at 81% long-term coverage preventing 13% (7–22) of infections overall, slightly lower because our counterfactual scenario has an estimated 52% long-term ART coverage. It is important to note that while these other studies explore the impact of PrEP and TasP under many coverage and adherence scenarios, our analysis estimates long-term coverage based on either observed or plausible increases in uptake, retention, and adherence. Nevertheless, the comparable results between all studies underlines the robustness and generalizability of the conclusions, that is, if FSW have PrEP and TasP adherence and retention as observed in Cotonou, the population-level impact of TasP is higher.

### Strengths and Limitations

There are several strengths to our modeling analysis. First, we accessed several rounds of historical cross-sectional data among several risk groups, including IBBS among pFSW and their clients from 1993–2017,^16,17,20,21,26,30–34^ and a FSW mapping study in Cotonou in 2012,^27^ informing demographic and sexual behavior parameters and HIV prevalence estimates over time for all groups. Our modeled trajectories reflected empirical data from Cotonou and therefore were an accurate representation of its demography and HIV epidemiology. Other data were used for cross-validation for further confidence (see Section 5, Supplemental Digital Content 1, http://links.lww.com/QAI/B549). Second, the PrEP/TasP study data provided estimates for key parameters including uptake, retention, and individual biological measures of adherence every 6 months: blood plasma tenofovir concentration for PrEP and viral load for TasP.^25,28^ Accounting for outreach, adherence counseling, migration, leaving sex work, and other legal and structural barriers to PrEP/TasP use among pFSW, our analysis is significantly more realistic compared with previous studies that did not reflect such data.^13,72,74–77^

Limitations to our analysis are mostly due to data uncertainties. Self-reported data used to inform our sexual behavior parameters may have been subject to social desirability or recall biases, although these would be mitigated by our use of confidential polling booth survey data^45^ and by assigning wide prior parameter ranges capturing uncertainty. We fixed adherence levels over 20 years in PrEP extension/scale-ups, which ignores the observed decrease in adherence over the 2-year study. However, this is justifiable because an extended PrEP intervention would allow more new initiations and dropouts, potentially maintaining a stable overall adherence pattern. There remains substantial uncertainty about the efficacy of PrEP for women, especially at intermediate adherence levels, based on clinical trial data and low biological availability of tenofovir in vaginal tissue.^9,78^ We performed sensitivity analyses (see Section 9, Supplemental Digital Content 1, http://links.lww.com/QAI/B549) showing that this uncertainty did not influence impact substantially because intermediate adherence represented only 17% of samples. The deterministic compartmental transmission model used cannot capture fine-grained individual-level behaviors but was appropriate for measuring the population-level impact. By capturing indirect HIV transmissions in our model, PrEP and TasP in pFSW incurred measurable protection in clients and the overall population, providing the full impact of these interventions.

## CONCLUSIONS

Our model analysis suggests that the 2-year PrEP/TasP study had a modest impact among pFSW and overall in Cotonou over 2 and 20 years. The observed migration and poor adherence were key barriers to high PrEP coverage and effectiveness, as such the TasP arm was substantially more impactful than the PrEP arm in reducing HIV infections and mortality. Indeed, achieving high PrEP coverage over 20 years is only possible with very increased PrEP initiation, and it would still prevent fewer infections and gained fewer life-years overall than a TasP extension, even with perfect PrEP adherence. In resource-limited circumstances, this analysis suggests that scaling up test-and-treat is a more effective population-level strategy in the HIV prevention toolkit,^79^ particularly among key populations, for both preventing direct and indirect transmissions and increasing survival. PrEP should be made available where possible within combination prevention strategies to improve individual choices, but strong efforts should be made to ensure good adherence and retention, particularly in highly vulnerable groups, such as FSW. Future work includes estimating the cost-effectiveness of PrEP and TasP strategies.
